# Correction: Argenziano, M., et al. The Endocannabinoid System in Pediatric Inflammatory and Immune Diseases. *Int. J. Mol. Sci.* 2019, *20*, 5875

**DOI:** 10.3390/ijms21082757

**Published:** 2020-04-15

**Authors:** Maura Argenziano, Chiara Tortora, Giulia Bellini, Alessandra Di Paola, Francesca Punzo, Francesca Rossi

**Affiliations:** 1 Department of Experimental Medicine, University of Campania Luigi Vanvitelli, 80138 Naples, Italy; maura.argenziano@unicampania.it (M.A.); giulia.bellini@uniroma3.it (G.B.); alessandra.dipaola@unicampania.it (A.D.P.); francesca.punzo19@gmail.com (F.P.); 2 Department of Women, Child, and General and Specialized Surgery, University of Campania Luigi Vanvitelli, 80138 Naples, Italy; chiara.tortora@unicampania.it

The authors wish to make the following corrections to this paper [1]:

Due to a lapse, the Funding section of the original version of the manuscript did not report the funding received to publish it [1].

The publishing of this paper was funded by “VALERE 2018–2019”

The authors would like to apologize for any inconvenience caused to the readers by these changes.
